# *cis*,*fac*-Dichlorido{*N*-[3,5-di-*tert*-butyl-2-(trimethyl­silyl­oxy)benz­yl]-*N*,*N*-bis­(2-pyridylmeth­yl)amine}(dimethyl sulfoxide)ruthenium(II) dichloro­methane disolvate

**DOI:** 10.1107/S1600536809041324

**Published:** 2009-10-17

**Authors:** Paul J. Fischer, Stefan G. Minasian, John Arnold

**Affiliations:** a Chemistry Department, Macalester College, 1600 Grand Avenue, Saint Paul, MN 55105, USA; b Chemistry Department, University of California, Berkeley, CA 94720-1460, USA

## Abstract

Reaction of dichloridotetra­kis(dimethyl sulfoxide)ruthenium(II) and *N*-[3,5-di-*tert*-butyl-2-(trimethyl­silyl­oxy)benz­yl]-*N*,*N*-bis­(2-pyridylmeth­yl)amine (BPPA-TMS) affords the thermodynamic product *cis*,*fac*-[RuCl_2_(BPPA-TMS)(DMSO)] and kinetic product *trans*,*mer*-[RuCl_2_(BPPA-TMS)(DMSO)]. The title complex, [RuCl_2_(C_30_H_43_N_3_OSi)(C_2_H_6_OS)]·2CH_2_Cl_2_, crystallizes as a dichloro­methane disolvate, with two formula units in the asymmetric unit. The complex exhibits a distorted-octa­hedral geometry about the low spin *d* ^6^ Ru^II^ center. The BPPA-TMS ligand is coordinated in a facial fashion, with the DMSO ligand *cis* to the aliphatic nitro­gen atom of the BPPA-TMS ligand. One of the two dichloromethane solvate molecules is disordered over two positions in a 0.695:0.305 ratio.

## Related literature

The application of tetra­dentate monoanionic (TDMA) ligands for stabilizing reactive metal complexes motivates ligand design efforts (Chomitz & Arnold, 2009 ▶). The TDMA precursor *N*,*N*-bis-(2-pyridylmeth­yl)(2-hydr­oxy-3,5-di-*tert*-butyl­benz­yl)amine (HBPPA) has been employed to prepare main group, transition metal and actinide BPPA complexes (Chomitz *et al.*, 2007 ▶; Marinescu *et al.*, 2007 ▶) but Ru(BPPA) complexes have not been reported. Coordination complexes of ruthenium that contain chlorido, DMSO, and pyridylic ligands demonstrate promising applications as chemotherapeutic agents (Velders *et al.*, 2004 ▶; Bratsos *et al.*, 2007 ▶). Substitution mechanisms for related complexes have been studied, see: Mola *et al.* (2007 ▶). 
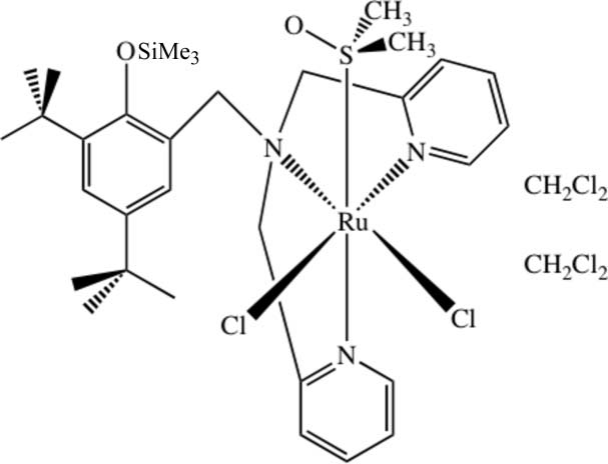


## Experimental

### 

#### Crystal data

[RuCl_2_(C_30_H_43_N_3_OSi)(C_2_H_6_OS)]·2CH_2_Cl_2_
*M* *_r_* = 909.71
Triclinic, 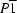

*a* = 10.913 (2) Å
*b* = 18.435 (4) Å
*c* = 22.413 (5) Å
α = 82.039 (3)°
β = 76.212 (3)°
γ = 89.148 (3)°
*V* = 4336.4 (15) Å^3^
*Z* = 4
Mo *K*α radiation
μ = 0.84 mm^−1^
*T* = 126 K
0.33 × 0.31 × 0.02 mm

#### Data collection

Bruker SMART CCD area-detector diffractometer
Absorption correction: multi-scan (*SADABS*; Bruker, 2001 ▶) *T*_min_ = 0.769, *T*_max_ = 0.988
52307 measured reflections
15775 independent reflections
8958 reflections with *I* > 2σ(*I*)
*R*_int_ = 0.085

#### Refinement

*R*[*F* ^2^ > 2σ(*F* ^2^)] = 0.049
*wR*(*F* ^2^) = 0.124
*S* = 0.98
15775 reflections
861 parameters
24 restraints
H-atom parameters constrained
Δρ_max_ = 0.70 e Å^−3^
Δρ_min_ = −0.57 e Å^−3^

### 

Data collection: *SMART* (Bruker, 2001 ▶); cell refinement: *SAINT* (Bruker, 2001 ▶); data reduction: *SAINT*; program(s) used to solve structure: *SHELXS97* (Sheldrick, 2008 ▶); program(s) used to refine structure: *SHELXL97* (Sheldrick, 2008 ▶); molecular graphics: *SHELXTL* (Sheldrick, 2008 ▶); software used to prepare material for publication: *SHELXTL* and *PLATON* (Spek, 2009 ▶).

## Supplementary Material

Crystal structure: contains datablocks I. DOI: 10.1107/S1600536809041324/bt5088sup1.cif

Structure factors: contains datablocks I. DOI: 10.1107/S1600536809041324/bt5088Isup2.hkl

Additional supplementary materials:  crystallographic information; 3D view; checkCIF report

## Figures and Tables

**Fig. 1.**
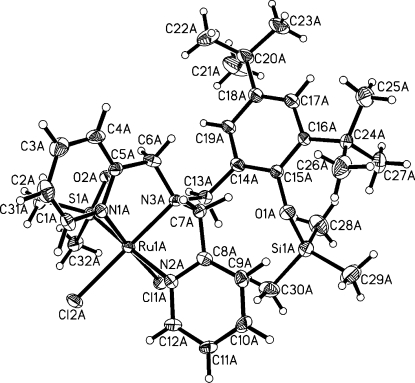
Molecular structure of one of the two molecules in the asymmetric unit of 1 (50% displacement ellipsoids). The solvent molecules are not shown.

## supplementary crystallographic information

### Comment

Desilylation of 1 to eliminate the trimethylsilyl substituent with concomitant Ru—O bond formation (and a tetradentate BPPA ligand) has been unsuccessful to date. The utility of BPPA-TMS as a BPPA transfer agent remains an open question.

The distorted octahedral structures at the low spin d^6^ Ru(II) centers of 1 and *cis*,*fac*-[RuCl_2_(BPEA)(DMSO)] (3, BPEA = *N*,*N*-bis(2-pyridylmethyl)ethylamine)) (Mola *et al.*, 2007) are very similar. The sulfur-bound DMSO ligand is *cis* to the aliphatic nitrogen in both 1 and 3, and the facial binding of the pyridyl and tertiary amine N atoms is seemingly independent tertiary nitrogen steric bulk (*i.e.*, ethyl (3) *versus* 2-trimethylsiloxo-3,5-*tert*- butylbenzyl (1)). The average Ru-pyridyl nitrogen lengths (1, 2.077 (3) Å; 3, 2.07 (4) Å) and the average Ru—Cl distances (1, 2.430 (6) Å; 3, 2.435 (9) Å) are statistically indistinguishable. The angles that define the edges of the distorted octahedra range from 79.61 (5) to 99.79 (4)° in 3 to 77.42 (14) to 99.43 (11) in 1. In both structures, the tertiary amine nitrogen-Ru—S angle (defined by N(3 A)—Ru(1 A)—S(1 A) in 1) is most obtuse while angles that define edges occupied by the facially bound ligands are most acute.

The ^1^H NMR spectra of 1 has similar features to that of 3, and is fully consistent with the solid-state structure. It is noteworthy that the six N—CH_2_-py and N—CH_2_—Ph ligand backbone H atoms are diastereotopic; each exhibits a doublet due to geminal coupling.

### Experimental

The following procedures were conducted under nitrogen using standard techniques for handling air and moisture sensitive substances. Ethanol (40 ml) was added to BPPA-TMS (1.50 g, 3.06 mmol) and RuCl_2_(DMSO)_4_ (1.48 g, 3.06 mmol). The mixture was heated to reflux (15 hr); the solids dissolved affording a red-orange solution. Solvent removal *in vacuo* provided a red oily residue that was washed with pentane (10 ml). Treatment with Et_2_O (45 ml) resulted in a yellow solid containing 1 and 2. The suspension was vigorously stirred (20 min) to generate a fine powder. The Et_2_O was decanted, and the solid was dried *in vacuo*. Kinetic product 2 was extracted with toluene (30 ml); the remaining solid was washed with toluene (10 ml) to remove traces of 2. The residue was dried *in vacuo*, and 1 was extracted with CH_2_Cl_2_ (30 ml). The CH_2_Cl_2_ extract was filtered, and the solvent removed *in vacuo* revealing yellow-orange 1. Pentane diffusion into a dichloromethane solution of 1 yielded crystals of 1 with 2 CH_2_Cl_2_ molecules per formula unit (1.13 g, 40%). Mp: 162–163 °C (dec).

### Refinement

All hydrogen atoms were placed in ideal positions with C-H ranging from 0.95Å to 0.99Å and U(H) set to 1.2U_eq_(C) or 1.5U_eq_(C_methyl_) and refined as riding atoms. Two dichloromethane molecules were multipositioned. These could not be modeled as anisotropic, partial molecules and their contribution to the scattering power was suppressed using the *SQUEEZE* option in *PLATON* (Spek, 2003). One of the two remaining dichloromethane solvents refined well while the other was split into two fragments refining to a 0.693 (16):0.307 (16) ratio. The final refinement employed 24 bond distance (SAME) and anisotropic (DELU) restraints.

### Crystal data

**Table:**

[RuCl_2_(C_30_H_43_N_3_OSi)(C_2_H_6_OS)]·2CH_2_Cl_2_	*Z* = 4
*M**_r_* = 909.71	*F*(000) = 1880
Triclinic, *P*1	*D*_x_ = 1.393 Mg m^−^^3^
Hall symbol: -P 1	Melting point: 162 K
*a* = 10.913 (2) Å	Mo *K*α radiation, λ = 0.71073 Å
*b* = 18.435 (4) Å	Cell parameters from 7479 reflections
*c* = 22.413 (5) Å	θ = 2.5–23.0°
α = 82.039 (3)°	µ = 0.84 mm^−^^1^
β = 76.212 (3)°	*T* = 126 K
γ = 89.148 (3)°	Plate, yellow
*V* = 4336.4 (15) Å^3^	0.33 × 0.31 × 0.02 mm

### Data collection

**Table:**

Bruker SMART CCD area-detector diffractometer	15775 independent reflections
Radiation source: sealed tube	8958 reflections with *I* > 2σ(*I*)
graphite	*R*_int_ = 0.085
φ and ω scans	θ_max_ = 25.4°, θ_min_ = 2.5°
Absorption correction: multi-scan (*SADABS*; Bruker, 2001)	*h* = −12→13
*T*_min_ = 0.769, *T*_max_ = 0.988	*k* = −21→22
52307 measured reflections	*l* = 0→26

### Refinement

**Table:**

Refinement on *F*^2^	Primary atom site location: structure-invariant direct methods
Least-squares matrix: full	Secondary atom site location: difference Fourier map
*R*[*F*^2^ > 2σ(*F*^2^)] = 0.049	Hydrogen site location: inferred from neighbouring sites
*wR*(*F*^2^) = 0.124	H-atom parameters constrained
*S* = 0.98	*w* = 1/[σ^2^(*F*_o_^2^) + (0.0583*P*)^2^] where *P* = (*F*_o_^2^ + 2*F*_c_^2^)/3
15775 reflections	(Δ/σ)_max_ = 0.001
861 parameters	Δρ_max_ = 0.70 e Å^−^^3^
24 restraints	Δρ_min_ = −0.57 e Å^−^^3^

### Special details

**Table:**

Experimental. Anal Calcd. For C_34_H_53_Cl_6_N_3_O_2_SSiRu C, 44.89; H, 5.87; N, 4.62. Found C, 45.13; H, 5.86; N, 4.96.NMR (CD_3_CN, 400 MHz) ^1^H δ 9.73 (d, J = 5.2 Hz 1H, α-pyridyl), 9.27 (d, J = 5.2, 1H, α-pyridyl), 7.57 (app t, J = 7.6 Hz, 1H, *para*-pyridyl), 7.44 (app t, J = 7.6 Hz, 1H, *para*-pyridyl), 7.37 (d, J = 2.4, 1H, aromatic phenol), 7.24 (app t, J = 6.0 Hz, 1H, B-pyridyl), 7.18 (d, J = 2.4 Hz, 1H, aromatic phenol), 7.01 (app t, J = 6.4 Hz, 1H, B-pyridyl), 6.94 (m, 2H, pyridyl), 5.27 (s, 4H, CH_2_Cl_2_), 5.05 (d, J = 14 Hz, 1H, NCH_2_py), 4.95 (d, J = 15 Hz, 1H, NCH_2_py), 4.69 (d, J = 14 Hz, 1H, NCH_2_py), 4.56 (d, J = 16 Hz, 1H, NCH_2_py), 3.62 (s, 3H, DMSO), 3.28 (d, J = 15 Hz, 1H, NCH_2_Ph), 3.11 (d, J = 17 Hz, NCH_2_Ph), 2.87 (s, 3H, DMSO), 1.41 (sm, 9H, *tert*-Bu), 1.29 (s, 9H, *tert*-Bu), 0.156 (s, 9H, SiMe_3_).NMR (CD_3_CN, 400 MHz) ^13^C{^1^H} δ 164.3, 160.9, 154.5, 153.2, 151.6, 143.0, 140.6, 136.5, 135.0, 129.3, 125.5, 123.7, 123.6, 123.5, 120.6, 119.6, 68.7, 65.1, 63.8, 53.6, 44.8, 44.0, 35.4, 34.2, 31.6, 31.4, 2.1. The expected sixteen aromatic carbon environments are present, along with eleven other environments (including one for for dichloromethane solvate), consistent with the solid-state structure of 1.
Geometry. All e.s.d.'s (except the e.s.d. in the dihedral angle between two l.s. planes) are estimated using the full covariance matrix. The cell e.s.d.'s are taken into account individually in the estimation of e.s.d.'s in distances, angles and torsion angles; correlations between e.s.d.'s in cell parameters are only used when they are defined by crystal symmetry. An approximate (isotropic) treatment of cell e.s.d.'s is used for estimating e.s.d.'s involving l.s. planes.
Refinement. A crystal (approximate dimensions 1/4*x* 0.12 *x* 0.04 mm^3^) was placed onto the tip of a 0.1 mm diameter glass capillary and mounted on a CCD area detector diffractometer for a data collection at 173 (2) K (*SMART* V5.054, Bruker Analytical X-ray Systems, Madison, WI (2001).). A preliminary set of cell constants was calculated from reflections harvested from three sets of 20 frames. These initial sets of frames were oriented such that orthogonal wedges of reciprocal space were surveyed. This produced initial orientation matrices determined from 48 reflections. The data collection was carried out using Mo *K*α radiation (graphite monochromator) with a frame time of 8 s and a detector distance of 4.8 cm. A randomly oriented region of reciprocal space was surveyed to the extent of one sphere and to a resolution of 0.77 Å. Four major sections of frames were collected with 0.30° steps in ω at four different φ settings and a detector position of -28° in 2θ. The intensity data were corrected for absorption and decay (*SADABS*). Refinement of *F*^2^ against ALL reflections. The weighted *R*-factor *wR* and goodness of fit *S* are based on *F*^2^, conventional *R*-factors *R* are based on *F*, with *F* set to zero for negative *F*^2^. The threshold expression of *F*^2^ > σ(*F*^2^) is used only for calculating *R*-factors(gt) *etc*. and is not relevant to the choice of reflections for refinement. *R*-factors based on *F*^2^ are statistically about twice as large as those based on *F*, and *R*- factors based on ALL data will be even larger.The structure is P-1 with two complexes and four dichloromethane molecules per asymmetric unit. The two indpendent ruthenium complexes are enantiomeric. The structure is best described as having *Z*=4 and *Z*'=2. The two ruthenium complexes are related by a pseudo-b-glide perpendicular to the *a* axis. The fit is quite good for the atoms bound to the Ru atom, but the atoms on the benzyl portion are not as good. However, this is just pseudosymmetry.The four dichlormethane molecules are related similarly. Two dichloromethane molecules were multipositioned. These could not be modeled as anisotropic, partial molecules. These two were commented out of the res file and *PLATON*/Squeeze was run to remove diffuse scattering effects from the diffraction data.*PLATON* / Squeeze results (Spek, 2009):Total Potential Solvent Area Vol: 608.2 Åper Unit Cell Vol: 4336.4 Å [14.0%]Total Potential Solvent Accessible Void Vol: 608.2 ÅElectron Count / Cell = 67 - To be included in D(calc), F000 & Mol.Wght.Correction - Since two DCM with electron count = 84 were removed, then 84 electron will be added into F000. The empirical formula is correct.One of the two remaining dichloromethane solvents refined well while the other was split into two fragments refining to a 0.695:0.305 ratio. The final refinement employed 24 bond distance (SAME) and anisotropic (DELU) restraints.

### Fractional atomic coordinates and isotropic or equivalent isotropic displacement parameters (Å^2^)

**Table:**

	*x*	*y*	*z*	*U*_iso_*/*U*_eq_	Occ. (<1)
Ru1A	0.29376 (4)	0.13565 (2)	0.115631 (17)	0.01704 (11)	
Cl1A	0.30575 (12)	0.24252 (6)	0.16712 (5)	0.0241 (3)	
Cl2A	0.34173 (12)	0.21477 (7)	0.01658 (5)	0.0265 (3)	
S1A	0.08900 (11)	0.15552 (7)	0.12116 (6)	0.0208 (3)	
Si1A	0.49345 (14)	0.11139 (8)	0.33698 (7)	0.0304 (4)	
O1A	0.4449 (3)	0.03571 (17)	0.31556 (14)	0.0276 (9)	
O2A	−0.0055 (3)	0.12446 (18)	0.17877 (14)	0.0271 (9)	
N1A	0.2767 (4)	0.04179 (19)	0.07772 (17)	0.0190 (9)	
N2A	0.4846 (3)	0.1227 (2)	0.11801 (16)	0.0182 (9)	
N3A	0.2778 (4)	0.0513 (2)	0.19379 (17)	0.0179 (9)	
C1A	0.3088 (5)	0.0334 (3)	0.0179 (2)	0.0254 (12)	
H1AA	0.3552	0.0716	−0.0112	0.031*	
C2A	0.2771 (5)	−0.0283 (3)	−0.0033 (2)	0.0342 (14)	
H2AB	0.3019	−0.0321	−0.0463	0.041*	
C3A	0.2101 (5)	−0.0843 (3)	0.0369 (3)	0.0353 (14)	
H3AA	0.1853	−0.1263	0.0222	0.042*	
C4A	0.1794 (5)	−0.0784 (3)	0.0994 (2)	0.0302 (13)	
H4AA	0.1357	−0.1174	0.1286	0.036*	
C5A	0.2133 (4)	−0.0146 (3)	0.1192 (2)	0.0227 (12)	
C6A	0.1828 (5)	−0.0024 (3)	0.1866 (2)	0.0236 (12)	
H6AA	0.0967	0.0169	0.1986	0.028*	
H6AB	0.1869	−0.0493	0.2136	0.028*	
C7A	0.4031 (4)	0.0129 (2)	0.1856 (2)	0.0188 (11)	
H7AA	0.4122	−0.0126	0.2262	0.023*	
H7AB	0.4065	−0.0241	0.1573	0.023*	
C8A	0.5079 (5)	0.0677 (2)	0.1596 (2)	0.0198 (11)	
C9A	0.6227 (5)	0.0616 (3)	0.1757 (2)	0.0240 (12)	
H9AA	0.6372	0.0219	0.2049	0.029*	
C10A	0.7160 (5)	0.1139 (3)	0.1486 (2)	0.0292 (13)	
H10A	0.7957	0.1106	0.1589	0.035*	
C11A	0.6914 (4)	0.1714 (3)	0.1061 (2)	0.0252 (13)	
H11B	0.7538	0.2083	0.0872	0.030*	
C12A	0.5754 (4)	0.1742 (3)	0.0919 (2)	0.0238 (12)	
H12B	0.5587	0.2134	0.0629	0.029*	
C13A	0.2423 (4)	0.0793 (2)	0.2559 (2)	0.0198 (11)	
H13A	0.1617	0.1056	0.2589	0.024*	
H13B	0.3077	0.1153	0.2572	0.024*	
C14A	0.2280 (5)	0.0210 (3)	0.3121 (2)	0.0235 (12)	
C15A	0.3303 (4)	−0.0031 (3)	0.3377 (2)	0.0198 (11)	
C16A	0.3174 (5)	−0.0649 (3)	0.3827 (2)	0.0223 (12)	
C17A	0.1948 (5)	−0.0944 (3)	0.4064 (2)	0.0277 (13)	
H17A	0.1837	−0.1358	0.4377	0.033*	
C18A	0.0890 (5)	−0.0676 (3)	0.3876 (2)	0.0265 (13)	
C19A	0.1088 (5)	−0.0115 (3)	0.3380 (2)	0.0233 (12)	
H19A	0.0397	0.0053	0.3213	0.028*	
C20A	−0.0437 (5)	−0.0989 (3)	0.4162 (3)	0.0395 (15)	
C21A	−0.1305 (6)	−0.0365 (4)	0.4392 (3)	0.073 (2)	
H21A	−0.2174	−0.0553	0.4547	0.109*	
H21B	−0.1268	0.0031	0.4048	0.109*	
H21C	−0.1022	−0.0177	0.4726	0.109*	
C22A	−0.0947 (6)	−0.1316 (4)	0.3685 (3)	0.079 (3)	
H22A	−0.0355	−0.1676	0.3504	0.119*	
H22B	−0.1053	−0.0927	0.3357	0.119*	
H22C	−0.1765	−0.1557	0.3885	0.119*	
C23A	−0.0488 (5)	−0.1573 (3)	0.4723 (3)	0.0505 (18)	
H23A	−0.0014	−0.2001	0.4588	0.076*	
H23B	−0.1368	−0.1719	0.4917	0.076*	
H23C	−0.0114	−0.1374	0.5024	0.076*	
C24A	0.4293 (5)	−0.1028 (3)	0.4046 (2)	0.0287 (13)	
C25A	0.3952 (6)	−0.1816 (3)	0.4386 (3)	0.0517 (18)	
H25A	0.3609	−0.2100	0.4123	0.078*	
H25B	0.3321	−0.1795	0.4776	0.078*	
H25C	0.4712	−0.2051	0.4476	0.078*	
C26A	0.5413 (5)	−0.1092 (3)	0.3498 (3)	0.0465 (16)	
H26A	0.5748	−0.0602	0.3309	0.070*	
H26B	0.5138	−0.1337	0.3190	0.070*	
H26C	0.6074	−0.1379	0.3645	0.070*	
C27A	0.4694 (5)	−0.0597 (3)	0.4507 (2)	0.0417 (15)	
H27A	0.4961	−0.0100	0.4304	0.063*	
H27B	0.5397	−0.0843	0.4648	0.063*	
H27C	0.3981	−0.0571	0.4864	0.063*	
C28A	0.3788 (6)	0.1316 (3)	0.4077 (2)	0.0467 (17)	
H28A	0.2999	0.1481	0.3972	0.070*	
H28B	0.4136	0.1701	0.4253	0.070*	
H28C	0.3622	0.0872	0.4383	0.070*	
C29A	0.6577 (5)	0.0943 (3)	0.3460 (3)	0.0551 (19)	
H29A	0.7100	0.0803	0.3075	0.083*	
H29B	0.6563	0.0547	0.3802	0.083*	
H29C	0.6929	0.1390	0.3550	0.083*	
C30A	0.5066 (5)	0.1913 (3)	0.2768 (2)	0.0393 (15)	
H30A	0.4221	0.2067	0.2732	0.059*	
H30B	0.5546	0.1783	0.2370	0.059*	
H30C	0.5503	0.2315	0.2880	0.059*	
C31A	0.0407 (5)	0.1256 (3)	0.0575 (2)	0.0300 (13)	
H31A	−0.0439	0.1435	0.0568	0.045*	
H31B	0.0396	0.0719	0.0623	0.045*	
H31C	0.1002	0.1449	0.0185	0.045*	
C32A	0.0529 (5)	0.2500 (2)	0.1080 (2)	0.0284 (13)	
H32A	−0.0375	0.2550	0.1099	0.043*	
H32B	0.1015	0.2717	0.0669	0.043*	
H32C	0.0747	0.2754	0.1398	0.043*	
Ru1B	0.09676 (4)	0.63569 (2)	0.115900 (18)	0.01900 (12)	
Cl1B	0.04663 (12)	0.74054 (6)	0.17174 (6)	0.0278 (3)	
Cl2B	0.11675 (13)	0.71495 (7)	0.01828 (6)	0.0323 (3)	
S1B	0.30095 (12)	0.65563 (7)	0.10974 (6)	0.0247 (3)	
Si1B	−0.21556 (15)	0.61342 (9)	0.37330 (7)	0.0355 (4)	
O1B	−0.1606 (3)	0.53386 (18)	0.34994 (15)	0.0296 (9)	
O2B	0.3538 (3)	0.62659 (19)	0.16344 (16)	0.0341 (9)	
N1B	0.1388 (4)	0.5427 (2)	0.07480 (18)	0.0227 (10)	
N2B	−0.0989 (4)	0.6230 (2)	0.13090 (18)	0.0219 (10)	
N3B	0.0615 (3)	0.5517 (2)	0.19497 (17)	0.0185 (9)	
C1B	0.1459 (5)	0.5359 (3)	0.0146 (2)	0.0268 (13)	
H1BA	0.1197	0.5755	−0.0112	0.032*	
C2B	0.1902 (5)	0.4731 (3)	−0.0107 (2)	0.0334 (14)	
H2BB	0.1956	0.4701	−0.0532	0.040*	
C3B	0.2263 (5)	0.4147 (3)	0.0269 (2)	0.0348 (14)	
H3BA	0.2582	0.3716	0.0104	0.042*	
C4B	0.2153 (5)	0.4203 (3)	0.0897 (2)	0.0277 (13)	
H4BA	0.2376	0.3805	0.1166	0.033*	
C5B	0.1715 (4)	0.4846 (3)	0.1120 (2)	0.0220 (12)	
C6B	0.1608 (4)	0.4966 (2)	0.1781 (2)	0.0205 (11)	
H6BA	0.1382	0.4499	0.2061	0.025*	
H6BB	0.2427	0.5145	0.1827	0.025*	
C7B	−0.0641 (4)	0.5167 (2)	0.1998 (2)	0.0212 (12)	
H7BA	−0.0519	0.4752	0.1755	0.025*	
H7BB	−0.1013	0.4968	0.2437	0.025*	
C8B	−0.1535 (5)	0.5687 (3)	0.1771 (2)	0.0209 (12)	
C9B	−0.2821 (5)	0.5614 (3)	0.1982 (2)	0.0313 (13)	
H9BA	−0.3181	0.5234	0.2306	0.038*	
C10B	−0.3592 (5)	0.6103 (3)	0.1717 (3)	0.0367 (15)	
H10B	−0.4485	0.6055	0.1852	0.044*	
C11B	−0.3037 (5)	0.6664 (3)	0.1252 (3)	0.0359 (14)	
H11A	−0.3541	0.7011	0.1069	0.043*	
C12B	−0.1740 (5)	0.6704 (3)	0.1064 (2)	0.0275 (13)	
H12A	−0.1360	0.7085	0.0745	0.033*	
C13B	0.0659 (5)	0.5802 (3)	0.25443 (19)	0.0225 (12)	
H13C	0.1443	0.6099	0.2470	0.027*	
H13D	−0.0062	0.6131	0.2650	0.027*	
C14B	0.0617 (5)	0.5227 (3)	0.3090 (2)	0.0230 (12)	
C15B	−0.0486 (5)	0.4984 (3)	0.3533 (2)	0.0250 (12)	
C16B	−0.0451 (5)	0.4371 (3)	0.3990 (2)	0.0276 (13)	
C17B	0.0722 (5)	0.4081 (3)	0.4004 (2)	0.0311 (14)	
H17B	0.0757	0.3676	0.4311	0.037*	
C18B	0.1847 (5)	0.4341 (3)	0.3601 (2)	0.0294 (13)	
C19B	0.1757 (5)	0.4917 (3)	0.3149 (2)	0.0287 (13)	
H19B	0.2510	0.5110	0.2866	0.034*	
C20B	0.3134 (5)	0.3992 (3)	0.3607 (3)	0.0381 (15)	
C21B	0.3074 (7)	0.3400 (4)	0.4148 (3)	0.089 (3)	
H21D	0.3919	0.3204	0.4133	0.134*	
H21E	0.2501	0.3005	0.4126	0.134*	
H21F	0.2764	0.3604	0.4536	0.134*	
C22B	0.3627 (8)	0.3685 (5)	0.3007 (3)	0.102 (3)	
H22D	0.4485	0.3508	0.2988	0.153*	
H22E	0.3640	0.4070	0.2656	0.153*	
H22F	0.3078	0.3278	0.2986	0.153*	
C23B	0.4075 (6)	0.4569 (4)	0.3655 (3)	0.074 (2)	
H23D	0.4914	0.4355	0.3609	0.111*	
H23E	0.3815	0.4740	0.4061	0.111*	
H23F	0.4101	0.4983	0.3326	0.111*	
C24B	−0.1653 (5)	0.4035 (3)	0.4473 (2)	0.0344 (14)	
C25B	−0.1391 (6)	0.3274 (3)	0.4791 (3)	0.0499 (18)	
H25D	−0.2166	0.3071	0.5082	0.075*	
H25E	−0.0736	0.3318	0.5017	0.075*	
H25F	−0.1105	0.2949	0.4477	0.075*	
C26B	−0.2744 (6)	0.3947 (3)	0.4168 (3)	0.0524 (18)	
H26D	−0.3490	0.3749	0.4485	0.079*	
H26E	−0.2504	0.3609	0.3860	0.079*	
H26F	−0.2939	0.4425	0.3964	0.079*	
C27B	−0.2033 (6)	0.4525 (3)	0.4985 (2)	0.0491 (18)	
H27D	−0.2791	0.4319	0.5289	0.074*	
H27E	−0.2208	0.5018	0.4802	0.074*	
H27F	−0.1343	0.4552	0.5193	0.074*	
C28B	−0.1051 (6)	0.6473 (3)	0.4147 (3)	0.0505 (17)	
H28D	−0.0215	0.6557	0.3864	0.076*	
H28E	−0.0994	0.6109	0.4501	0.076*	
H28F	−0.1359	0.6934	0.4295	0.076*	
C29B	−0.3792 (6)	0.5965 (3)	0.4213 (3)	0.0556 (18)	
H29D	−0.3771	0.5670	0.4610	0.083*	
H29E	−0.4279	0.5701	0.3993	0.083*	
H29F	−0.4190	0.6434	0.4290	0.083*	
C30B	−0.2306 (6)	0.6826 (3)	0.3077 (3)	0.0477 (16)	
H30D	−0.1471	0.6941	0.2799	0.072*	
H30E	−0.2667	0.7272	0.3234	0.072*	
H30F	−0.2860	0.6633	0.2848	0.072*	
C31B	0.4028 (5)	0.6244 (3)	0.0436 (2)	0.0397 (15)	
H31D	0.4893	0.6417	0.0395	0.059*	
H31E	0.3745	0.6436	0.0062	0.059*	
H31F	0.4005	0.5707	0.0489	0.059*	
C32B	0.3455 (5)	0.7505 (3)	0.0915 (2)	0.0321 (14)	
H32D	0.4363	0.7559	0.0879	0.048*	
H32E	0.2988	0.7771	0.1245	0.048*	
H32F	0.3259	0.7705	0.0520	0.048*	
Cl1C	0.47593 (17)	0.55631 (10)	0.87226 (9)	0.0723 (6)	
C1C	0.4109 (5)	0.6421 (2)	0.8598 (3)	0.068 (2)	
H1C1	0.3867	0.6620	0.8997	0.082*	
H1C2	0.4759	0.6755	0.8312	0.082*	
Cl2C	0.27935 (18)	0.64103 (10)	0.82890 (11)	0.0853 (7)	
Cl1D	−0.0460 (8)	0.3220 (2)	0.2756 (2)	0.075 (2)	0.693 (16)
C1D	0.0112 (11)	0.2334 (4)	0.2809 (3)	0.050 (3)	0.693 (16)
H1D1	−0.0374	0.2030	0.2611	0.060*	0.693 (16)
H1D2	0.1002	0.2347	0.2571	0.060*	0.693 (16)
Cl2D	0.0030 (13)	0.1918 (5)	0.3562 (4)	0.093 (2)	0.693 (16)
Cl1E	−0.0996 (13)	0.2932 (12)	0.2747 (6)	0.096 (4)	0.307 (16)
C1E	0.0426 (14)	0.2746 (15)	0.2952 (10)	0.075 (8)	0.307 (16)
H1E1	0.1067	0.2647	0.2580	0.090*	0.307 (16)
H1E2	0.0711	0.3187	0.3092	0.090*	0.307 (16)
Cl2E	0.035 (4)	0.2007 (13)	0.3534 (9)	0.109 (9)	0.307 (16)

### Atomic displacement parameters (Å^2^)

**Table:**

	*U*^11^	*U*^22^	*U*^33^	*U*^12^	*U*^13^	*U*^23^
Ru1A	0.0150 (2)	0.0158 (2)	0.0193 (2)	−0.00084 (17)	−0.00409 (18)	0.00129 (17)
Cl1A	0.0263 (7)	0.0180 (7)	0.0291 (7)	0.0011 (5)	−0.0106 (6)	−0.0010 (5)
Cl2A	0.0276 (8)	0.0246 (7)	0.0232 (7)	−0.0017 (6)	−0.0035 (6)	0.0065 (5)
S1A	0.0161 (7)	0.0228 (7)	0.0237 (7)	0.0007 (5)	−0.0067 (6)	0.0000 (6)
Si1A	0.0315 (9)	0.0294 (9)	0.0326 (9)	−0.0023 (7)	−0.0132 (7)	−0.0018 (7)
O1A	0.022 (2)	0.030 (2)	0.030 (2)	−0.0017 (16)	−0.0065 (16)	−0.0006 (16)
O2A	0.0152 (19)	0.035 (2)	0.0273 (19)	−0.0032 (16)	−0.0022 (16)	0.0034 (16)
N1A	0.020 (2)	0.013 (2)	0.025 (2)	0.0036 (17)	−0.0107 (19)	0.0009 (18)
N2A	0.018 (2)	0.019 (2)	0.017 (2)	0.0043 (18)	−0.0025 (18)	−0.0018 (18)
N3A	0.018 (2)	0.014 (2)	0.021 (2)	−0.0021 (17)	−0.0058 (18)	0.0002 (17)
C1A	0.027 (3)	0.028 (3)	0.019 (3)	0.006 (2)	−0.005 (2)	0.002 (2)
C2A	0.047 (4)	0.033 (4)	0.025 (3)	0.009 (3)	−0.010 (3)	−0.012 (3)
C3A	0.043 (4)	0.022 (3)	0.046 (4)	0.003 (3)	−0.020 (3)	−0.010 (3)
C4A	0.030 (3)	0.016 (3)	0.045 (4)	−0.004 (2)	−0.014 (3)	0.004 (2)
C5A	0.017 (3)	0.020 (3)	0.032 (3)	0.000 (2)	−0.012 (2)	0.003 (2)
C6A	0.023 (3)	0.017 (3)	0.026 (3)	0.000 (2)	0.001 (2)	0.001 (2)
C7A	0.017 (3)	0.014 (3)	0.027 (3)	0.005 (2)	−0.010 (2)	0.000 (2)
C8A	0.023 (3)	0.014 (3)	0.021 (3)	0.003 (2)	−0.002 (2)	−0.004 (2)
C9A	0.021 (3)	0.027 (3)	0.023 (3)	0.008 (2)	−0.007 (2)	−0.001 (2)
C10A	0.012 (3)	0.045 (4)	0.031 (3)	0.000 (2)	−0.004 (2)	−0.004 (3)
C11A	0.015 (3)	0.028 (3)	0.029 (3)	−0.003 (2)	0.001 (2)	−0.002 (2)
C12A	0.021 (3)	0.021 (3)	0.026 (3)	−0.004 (2)	0.000 (2)	0.000 (2)
C13A	0.017 (3)	0.020 (3)	0.022 (3)	0.003 (2)	−0.002 (2)	−0.004 (2)
C14A	0.027 (3)	0.018 (3)	0.024 (3)	0.000 (2)	−0.006 (2)	0.001 (2)
C15A	0.016 (3)	0.021 (3)	0.022 (3)	0.002 (2)	−0.004 (2)	0.000 (2)
C16A	0.026 (3)	0.017 (3)	0.024 (3)	−0.001 (2)	−0.011 (2)	0.002 (2)
C17A	0.032 (3)	0.027 (3)	0.022 (3)	−0.004 (3)	−0.006 (3)	0.002 (2)
C18A	0.025 (3)	0.028 (3)	0.025 (3)	−0.001 (2)	−0.006 (2)	0.001 (2)
C19A	0.023 (3)	0.023 (3)	0.021 (3)	0.000 (2)	−0.003 (2)	0.003 (2)
C20A	0.022 (3)	0.047 (4)	0.041 (4)	−0.008 (3)	−0.006 (3)	0.018 (3)
C21A	0.029 (4)	0.065 (5)	0.097 (6)	0.009 (3)	0.016 (4)	0.029 (4)
C22A	0.058 (5)	0.106 (6)	0.072 (5)	−0.058 (5)	−0.024 (4)	0.021 (5)
C23A	0.032 (4)	0.054 (4)	0.052 (4)	−0.012 (3)	−0.002 (3)	0.027 (3)
C24A	0.024 (3)	0.030 (3)	0.035 (3)	−0.003 (2)	−0.016 (3)	0.006 (2)
C25A	0.048 (4)	0.036 (4)	0.076 (5)	0.002 (3)	−0.034 (4)	0.011 (3)
C26A	0.034 (4)	0.050 (4)	0.060 (4)	0.011 (3)	−0.020 (3)	−0.009 (3)
C27A	0.045 (4)	0.048 (4)	0.039 (3)	0.001 (3)	−0.025 (3)	−0.002 (3)
C28A	0.061 (5)	0.043 (4)	0.035 (3)	0.006 (3)	−0.009 (3)	−0.007 (3)
C29A	0.041 (4)	0.045 (4)	0.085 (5)	−0.008 (3)	−0.032 (4)	0.003 (4)
C30A	0.049 (4)	0.026 (3)	0.040 (3)	−0.009 (3)	−0.006 (3)	−0.003 (3)
C31A	0.022 (3)	0.039 (3)	0.034 (3)	0.005 (2)	−0.014 (3)	−0.009 (3)
C32A	0.019 (3)	0.025 (3)	0.043 (3)	0.006 (2)	−0.014 (3)	0.000 (3)
Ru1B	0.0183 (2)	0.0168 (2)	0.0210 (2)	0.00028 (17)	−0.00551 (18)	0.00220 (17)
Cl1B	0.0285 (8)	0.0185 (7)	0.0332 (7)	0.0010 (5)	−0.0036 (6)	0.0000 (6)
Cl2B	0.0388 (9)	0.0288 (8)	0.0268 (7)	−0.0020 (6)	−0.0106 (6)	0.0105 (6)
S1B	0.0189 (7)	0.0261 (8)	0.0276 (7)	0.0002 (6)	−0.0048 (6)	0.0004 (6)
Si1B	0.0317 (10)	0.0375 (10)	0.0354 (9)	0.0037 (7)	−0.0036 (8)	−0.0065 (8)
O1B	0.031 (2)	0.028 (2)	0.029 (2)	0.0044 (17)	−0.0087 (17)	−0.0026 (16)
O2B	0.022 (2)	0.038 (2)	0.042 (2)	0.0010 (17)	−0.0139 (18)	0.0059 (18)
N1B	0.018 (2)	0.023 (2)	0.027 (2)	0.0021 (18)	−0.0056 (19)	−0.001 (2)
N2B	0.019 (2)	0.022 (3)	0.026 (2)	0.0008 (19)	−0.008 (2)	−0.005 (2)
N3B	0.013 (2)	0.020 (2)	0.023 (2)	−0.0010 (17)	−0.0072 (18)	0.0022 (18)
C1B	0.021 (3)	0.030 (3)	0.025 (3)	−0.005 (2)	−0.004 (2)	0.005 (2)
C2B	0.041 (4)	0.030 (3)	0.031 (3)	−0.003 (3)	−0.008 (3)	−0.009 (3)
C3B	0.031 (3)	0.029 (3)	0.042 (4)	−0.001 (3)	0.001 (3)	−0.013 (3)
C4B	0.024 (3)	0.020 (3)	0.036 (3)	0.001 (2)	−0.005 (3)	0.002 (2)
C5B	0.018 (3)	0.018 (3)	0.027 (3)	−0.002 (2)	−0.003 (2)	0.003 (2)
C6B	0.023 (3)	0.017 (3)	0.021 (3)	0.007 (2)	−0.008 (2)	0.004 (2)
C7B	0.018 (3)	0.019 (3)	0.024 (3)	−0.005 (2)	−0.005 (2)	0.004 (2)
C8B	0.021 (3)	0.020 (3)	0.025 (3)	−0.003 (2)	−0.010 (2)	−0.005 (2)
C9B	0.026 (3)	0.029 (3)	0.040 (3)	−0.002 (3)	−0.012 (3)	−0.001 (3)
C10B	0.016 (3)	0.043 (4)	0.054 (4)	0.001 (3)	−0.013 (3)	−0.010 (3)
C11B	0.032 (4)	0.033 (3)	0.048 (4)	0.006 (3)	−0.019 (3)	−0.007 (3)
C12B	0.028 (3)	0.021 (3)	0.034 (3)	0.006 (2)	−0.014 (3)	0.005 (2)
C13B	0.029 (3)	0.024 (3)	0.015 (3)	0.004 (2)	−0.007 (2)	−0.001 (2)
C14B	0.026 (3)	0.023 (3)	0.020 (3)	−0.001 (2)	−0.007 (2)	−0.002 (2)
C15B	0.021 (3)	0.024 (3)	0.031 (3)	0.002 (2)	−0.008 (2)	−0.001 (2)
C16B	0.037 (4)	0.025 (3)	0.021 (3)	−0.002 (3)	−0.008 (3)	−0.002 (2)
C17B	0.042 (4)	0.024 (3)	0.026 (3)	0.002 (3)	−0.008 (3)	0.002 (2)
C18B	0.033 (3)	0.027 (3)	0.028 (3)	0.005 (3)	−0.005 (3)	−0.004 (3)
C19B	0.031 (3)	0.035 (3)	0.021 (3)	−0.006 (3)	−0.007 (2)	−0.002 (2)
C20B	0.034 (4)	0.045 (4)	0.038 (4)	0.017 (3)	−0.015 (3)	−0.005 (3)
C21B	0.067 (6)	0.097 (7)	0.088 (6)	0.035 (5)	−0.018 (5)	0.040 (5)
C22B	0.102 (7)	0.138 (8)	0.089 (6)	0.079 (6)	−0.041 (5)	−0.072 (6)
C23B	0.044 (5)	0.076 (6)	0.108 (6)	0.022 (4)	−0.028 (4)	−0.015 (5)
C24B	0.033 (3)	0.033 (3)	0.030 (3)	−0.003 (3)	0.002 (3)	0.004 (3)
C25B	0.054 (4)	0.033 (4)	0.054 (4)	−0.011 (3)	−0.007 (3)	0.014 (3)
C26B	0.046 (4)	0.055 (4)	0.049 (4)	−0.019 (3)	−0.005 (3)	0.008 (3)
C27B	0.059 (5)	0.049 (4)	0.030 (3)	−0.004 (3)	0.008 (3)	−0.004 (3)
C28B	0.055 (4)	0.046 (4)	0.056 (4)	−0.001 (3)	−0.017 (3)	−0.018 (3)
C29B	0.044 (4)	0.056 (5)	0.059 (4)	0.008 (3)	0.005 (3)	−0.015 (3)
C30B	0.048 (4)	0.045 (4)	0.049 (4)	0.020 (3)	−0.010 (3)	−0.006 (3)
C31B	0.024 (3)	0.041 (4)	0.050 (4)	−0.004 (3)	0.005 (3)	−0.013 (3)
C32B	0.025 (3)	0.030 (3)	0.037 (3)	−0.005 (2)	−0.003 (3)	0.001 (3)
Cl1C	0.0637 (13)	0.0573 (12)	0.0960 (14)	−0.0096 (9)	−0.0320 (11)	0.0135 (10)
C1C	0.089 (6)	0.042 (4)	0.079 (5)	−0.016 (4)	−0.037 (5)	0.001 (4)
Cl2C	0.0590 (13)	0.0635 (13)	0.1423 (19)	0.0031 (10)	−0.0388 (13)	−0.0191 (13)
Cl1D	0.100 (5)	0.048 (2)	0.075 (2)	0.026 (2)	−0.015 (2)	−0.0104 (17)
C1D	0.065 (8)	0.042 (5)	0.044 (4)	0.013 (5)	−0.007 (5)	−0.017 (4)
Cl2D	0.137 (5)	0.073 (3)	0.054 (3)	0.031 (3)	−0.006 (3)	0.004 (3)
Cl1E	0.085 (8)	0.107 (12)	0.109 (7)	0.030 (7)	−0.040 (6)	−0.031 (7)
C1E	0.077 (14)	0.11 (2)	0.047 (15)	0.013 (18)	−0.022 (13)	−0.028 (11)
Cl2E	0.17 (2)	0.115 (13)	0.045 (8)	0.076 (13)	−0.028 (9)	−0.038 (7)

### Geometric parameters (Å, °)

**Table:**

Ru1A—N1A	2.060 (4)	S1B—O2B	1.487 (4)
Ru1A—N2A	2.105 (4)	S1B—C32B	1.787 (5)
Ru1A—N3A	2.151 (4)	S1B—C31B	1.788 (5)
Ru1A—S1A	2.2359 (14)	Si1B—O1B	1.680 (4)
Ru1A—Cl2A	2.4293 (13)	Si1B—C30B	1.846 (6)
Ru1A—Cl1A	2.4371 (12)	Si1B—C28B	1.850 (6)
S1A—O2A	1.496 (3)	Si1B—C29B	1.858 (6)
S1A—C32A	1.780 (5)	O1B—C15B	1.391 (6)
S1A—C31A	1.790 (5)	N1B—C1B	1.356 (6)
Si1A—O1A	1.670 (3)	N1B—C5B	1.361 (6)
Si1A—C30A	1.837 (5)	N2B—C12B	1.341 (6)
Si1A—C28A	1.850 (5)	N2B—C8B	1.366 (6)
Si1A—C29A	1.868 (6)	N3B—C6B	1.492 (6)
O1A—C15A	1.397 (5)	N3B—C7B	1.498 (5)
N1A—C1A	1.333 (6)	N3B—C13B	1.510 (5)
N1A—C5A	1.368 (5)	C1B—C2B	1.391 (7)
N2A—C8A	1.344 (6)	C1B—H1BA	0.9500
N2A—C12A	1.349 (5)	C2B—C3B	1.385 (7)
N3A—C6A	1.495 (5)	C2B—H2BB	0.9500
N3A—C13A	1.514 (5)	C3B—C4B	1.402 (7)
N3A—C7A	1.514 (6)	C3B—H3BA	0.9500
C1A—C2A	1.370 (6)	C4B—C5B	1.384 (6)
C1A—H1AA	0.9500	C4B—H4BA	0.9500
C2A—C3A	1.365 (7)	C5B—C6B	1.506 (6)
C2A—H2AB	0.9500	C6B—H6BA	0.9900
C3A—C4A	1.378 (7)	C6B—H6BB	0.9900
C3A—H3AA	0.9500	C7B—C8B	1.485 (7)
C4A—C5A	1.399 (6)	C7B—H7BA	0.9900
C4A—H4AA	0.9500	C7B—H7BB	0.9900
C5A—C6A	1.515 (6)	C8B—C9B	1.372 (7)
C6A—H6AA	0.9900	C9B—C10B	1.395 (7)
C6A—H6AB	0.9900	C9B—H9BA	0.9500
C7A—C8A	1.492 (6)	C10B—C11B	1.394 (7)
C7A—H7AA	0.9900	C10B—H10B	0.9500
C7A—H7AB	0.9900	C11B—C12B	1.377 (7)
C8A—C9A	1.383 (7)	C11B—H11A	0.9500
C9A—C10A	1.380 (6)	C12B—H12A	0.9500
C9A—H9AA	0.9500	C13B—C14B	1.495 (6)
C10A—C11A	1.393 (7)	C13B—H13C	0.9900
C10A—H10A	0.9500	C13B—H13D	0.9900
C11A—C12A	1.376 (6)	C14B—C19B	1.389 (7)
C11A—H11B	0.9500	C14B—C15B	1.400 (6)
C12A—H12B	0.9500	C15B—C16B	1.422 (7)
C13A—C14A	1.519 (6)	C16B—C17B	1.385 (7)
C13A—H13A	0.9900	C16B—C24B	1.561 (7)
C13A—H13B	0.9900	C17B—C18B	1.386 (7)
C14A—C19A	1.397 (6)	C17B—H17B	0.9500
C14A—C15A	1.411 (7)	C18B—C19B	1.383 (7)
C15A—C16A	1.399 (6)	C18B—C20B	1.538 (7)
C16A—C17A	1.404 (6)	C19B—H19B	0.9500
C16A—C24A	1.544 (7)	C20B—C21B	1.504 (8)
C17A—C18A	1.383 (7)	C20B—C22B	1.508 (8)
C17A—H17A	0.9500	C20B—C23B	1.521 (8)
C18A—C19A	1.387 (6)	C21B—H21D	0.9800
C18A—C20A	1.523 (7)	C21B—H21E	0.9800
C19A—H19A	0.9500	C21B—H21F	0.9800
C20A—C22A	1.513 (8)	C22B—H22D	0.9800
C20A—C23A	1.529 (7)	C22B—H22E	0.9800
C20A—C21A	1.545 (8)	C22B—H22F	0.9800
C21A—H21A	0.9800	C23B—H23D	0.9800
C21A—H21B	0.9800	C23B—H23E	0.9800
C21A—H21C	0.9800	C23B—H23F	0.9800
C22A—H22A	0.9800	C24B—C26B	1.529 (8)
C22A—H22B	0.9800	C24B—C27B	1.535 (7)
C22A—H22C	0.9800	C24B—C25B	1.537 (7)
C23A—H23A	0.9800	C25B—H25D	0.9800
C23A—H23B	0.9800	C25B—H25E	0.9800
C23A—H23C	0.9800	C25B—H25F	0.9800
C24A—C26A	1.528 (7)	C26B—H26D	0.9800
C24A—C27A	1.532 (7)	C26B—H26E	0.9800
C24A—C25A	1.551 (7)	C26B—H26F	0.9800
C25A—H25A	0.9800	C27B—H27D	0.9800
C25A—H25B	0.9800	C27B—H27E	0.9800
C25A—H25C	0.9800	C27B—H27F	0.9800
C26A—H26A	0.9800	C28B—H28D	0.9800
C26A—H26B	0.9800	C28B—H28E	0.9800
C26A—H26C	0.9800	C28B—H28F	0.9800
C27A—H27A	0.9800	C29B—H29D	0.9800
C27A—H27B	0.9800	C29B—H29E	0.9800
C27A—H27C	0.9800	C29B—H29F	0.9800
C28A—H28A	0.9800	C30B—H30D	0.9800
C28A—H28B	0.9800	C30B—H30E	0.9800
C28A—H28C	0.9800	C30B—H30F	0.9800
C29A—H29A	0.9800	C31B—H31D	0.9800
C29A—H29B	0.9800	C31B—H31E	0.9800
C29A—H29C	0.9800	C31B—H31F	0.9800
C30A—H30A	0.9800	C32B—H32D	0.9800
C30A—H30B	0.9800	C32B—H32E	0.9800
C30A—H30C	0.9800	C32B—H32F	0.9800
C31A—H31A	0.9800	Cl1C—C1C	1.738 (3)
C31A—H31B	0.9800	C1C—Cl2C	1.738 (3)
C31A—H31C	0.9800	C1C—H1C1	0.9900
C32A—H32A	0.9800	C1C—H1C2	0.9900
C32A—H32B	0.9800	Cl1D—C1D	1.739 (3)
C32A—H32C	0.9800	C1D—Cl2D	1.737 (4)
Ru1B—N1B	2.052 (4)	C1D—H1D1	0.9900
Ru1B—N2B	2.092 (4)	C1D—H1D2	0.9900
Ru1B—N3B	2.149 (4)	Cl1E—C1E	1.738 (4)
Ru1B—S1B	2.2324 (14)	C1E—Cl2E	1.738 (4)
Ru1B—Cl2B	2.4220 (13)	C1E—H1E1	0.9900
Ru1B—Cl1B	2.4320 (13)	C1E—H1E2	0.9900
			
N1A—Ru1A—N2A	96.38 (15)	S1B—Ru1B—Cl2B	89.29 (5)
N1A—Ru1A—N3A	77.42 (14)	N1B—Ru1B—Cl1B	175.87 (12)
N2A—Ru1A—N3A	79.17 (14)	N2B—Ru1B—Cl1B	84.96 (11)
N1A—Ru1A—S1A	88.67 (11)	N3B—Ru1B—Cl1B	97.72 (11)
N2A—Ru1A—S1A	174.29 (10)	S1B—Ru1B—Cl1B	89.06 (5)
N3A—Ru1A—S1A	99.43 (11)	Cl2B—Ru1B—Cl1B	90.20 (5)
N1A—Ru1A—Cl2A	94.58 (11)	O2B—S1B—C32B	106.0 (2)
N2A—Ru1A—Cl2A	93.85 (10)	O2B—S1B—C31B	105.7 (2)
N3A—Ru1A—Cl2A	168.59 (11)	C32B—S1B—C31B	97.9 (2)
S1A—Ru1A—Cl2A	88.38 (5)	O2B—S1B—Ru1B	118.80 (14)
N1A—Ru1A—Cl1A	176.16 (11)	C32B—S1B—Ru1B	112.96 (18)
N2A—Ru1A—Cl1A	84.67 (10)	C31B—S1B—Ru1B	113.15 (18)
N3A—Ru1A—Cl1A	99.19 (10)	O1B—Si1B—C30B	112.1 (2)
S1A—Ru1A—Cl1A	90.12 (4)	O1B—Si1B—C28B	107.8 (2)
Cl2A—Ru1A—Cl1A	89.03 (4)	C30B—Si1B—C28B	110.0 (3)
O2A—S1A—C32A	105.4 (2)	O1B—Si1B—C29B	107.9 (2)
O2A—S1A—C31A	106.6 (2)	C30B—Si1B—C29B	104.9 (3)
C32A—S1A—C31A	98.9 (2)	C28B—Si1B—C29B	114.1 (3)
O2A—S1A—Ru1A	118.72 (15)	C15B—O1B—Si1B	130.3 (3)
C32A—S1A—Ru1A	113.44 (16)	C1B—N1B—C5B	118.4 (4)
C31A—S1A—Ru1A	111.64 (17)	C1B—N1B—Ru1B	126.7 (3)
O1A—Si1A—C30A	112.6 (2)	C5B—N1B—Ru1B	114.7 (3)
O1A—Si1A—C28A	108.5 (2)	C12B—N2B—C8B	118.5 (4)
C30A—Si1A—C28A	108.7 (3)	C12B—N2B—Ru1B	125.0 (3)
O1A—Si1A—C29A	106.6 (2)	C8B—N2B—Ru1B	115.6 (3)
C30A—Si1A—C29A	105.4 (3)	C6B—N3B—C7B	108.0 (3)
C28A—Si1A—C29A	115.1 (3)	C6B—N3B—C13B	112.2 (4)
C15A—O1A—Si1A	129.6 (3)	C7B—N3B—C13B	111.2 (3)
C1A—N1A—C5A	118.2 (4)	C6B—N3B—Ru1B	104.3 (3)
C1A—N1A—Ru1A	127.2 (3)	C7B—N3B—Ru1B	107.8 (3)
C5A—N1A—Ru1A	114.2 (3)	C13B—N3B—Ru1B	113.0 (3)
C8A—N2A—C12A	118.9 (4)	N1B—C1B—C2B	122.2 (5)
C8A—N2A—Ru1A	115.5 (3)	N1B—C1B—H1BA	118.9
C12A—N2A—Ru1A	123.8 (3)	C2B—C1B—H1BA	118.9
C6A—N3A—C13A	112.6 (3)	C3B—C2B—C1B	119.1 (5)
C6A—N3A—C7A	107.0 (3)	C3B—C2B—H2BB	120.4
C13A—N3A—C7A	110.8 (4)	C1B—C2B—H2BB	120.4
C6A—N3A—Ru1A	105.0 (3)	C2B—C3B—C4B	119.1 (5)
C13A—N3A—Ru1A	113.8 (3)	C2B—C3B—H3BA	120.5
C7A—N3A—Ru1A	107.1 (3)	C4B—C3B—H3BA	120.5
N1A—C1A—C2A	122.3 (5)	C5B—C4B—C3B	119.0 (5)
N1A—C1A—H1AA	118.8	C5B—C4B—H4BA	120.5
C2A—C1A—H1AA	118.8	C3B—C4B—H4BA	120.5
C3A—C2A—C1A	120.6 (5)	N1B—C5B—C4B	122.2 (5)
C3A—C2A—H2AB	119.7	N1B—C5B—C6B	115.0 (4)
C1A—C2A—H2AB	119.7	C4B—C5B—C6B	122.8 (5)
C2A—C3A—C4A	118.5 (5)	N3B—C6B—C5B	109.1 (4)
C2A—C3A—H3AA	120.8	N3B—C6B—H6BA	109.9
C4A—C3A—H3AA	120.8	C5B—C6B—H6BA	109.9
C3A—C4A—C5A	119.2 (5)	N3B—C6B—H6BB	109.9
C3A—C4A—H4AA	120.4	C5B—C6B—H6BB	109.9
C5A—C4A—H4AA	120.4	H6BA—C6B—H6BB	108.3
N1A—C5A—C4A	121.1 (4)	C8B—C7B—N3B	112.6 (4)
N1A—C5A—C6A	115.5 (4)	C8B—C7B—H7BA	109.1
C4A—C5A—C6A	123.4 (4)	N3B—C7B—H7BA	109.1
N3A—C6A—C5A	107.8 (3)	C8B—C7B—H7BB	109.1
N3A—C6A—H6AA	110.1	N3B—C7B—H7BB	109.1
C5A—C6A—H6AA	110.1	H7BA—C7B—H7BB	107.8
N3A—C6A—H6AB	110.1	N2B—C8B—C9B	121.7 (5)
C5A—C6A—H6AB	110.1	N2B—C8B—C7B	115.1 (4)
H6AA—C6A—H6AB	108.5	C9B—C8B—C7B	123.1 (4)
C8A—C7A—N3A	109.6 (4)	C8B—C9B—C10B	119.2 (5)
C8A—C7A—H7AA	109.8	C8B—C9B—H9BA	120.4
N3A—C7A—H7AA	109.8	C10B—C9B—H9BA	120.4
C8A—C7A—H7AB	109.8	C11B—C10B—C9B	119.1 (5)
N3A—C7A—H7AB	109.8	C11B—C10B—H10B	120.4
H7AA—C7A—H7AB	108.2	C9B—C10B—H10B	120.4
N2A—C8A—C9A	122.1 (4)	C12B—C11B—C10B	118.4 (5)
N2A—C8A—C7A	115.4 (4)	C12B—C11B—H11A	120.8
C9A—C8A—C7A	122.5 (4)	C10B—C11B—H11A	120.8
C10A—C9A—C8A	119.1 (5)	N2B—C12B—C11B	123.0 (5)
C10A—C9A—H9AA	120.5	N2B—C12B—H12A	118.5
C8A—C9A—H9AA	120.5	C11B—C12B—H12A	118.5
C9A—C10A—C11A	118.9 (5)	C14B—C13B—N3B	115.2 (4)
C9A—C10A—H10A	120.5	C14B—C13B—H13C	108.5
C11A—C10A—H10A	120.5	N3B—C13B—H13C	108.5
C12A—C11A—C10A	119.0 (4)	C14B—C13B—H13D	108.5
C12A—C11A—H11B	120.5	N3B—C13B—H13D	108.5
C10A—C11A—H11B	120.5	H13C—C13B—H13D	107.5
N2A—C12A—C11A	122.0 (5)	C19B—C14B—C15B	118.8 (5)
N2A—C12A—H12B	119.0	C19B—C14B—C13B	116.8 (4)
C11A—C12A—H12B	119.0	C15B—C14B—C13B	124.4 (5)
N3A—C13A—C14A	115.2 (4)	O1B—C15B—C14B	118.4 (4)
N3A—C13A—H13A	108.5	O1B—C15B—C16B	121.6 (4)
C14A—C13A—H13A	108.5	C14B—C15B—C16B	120.0 (5)
N3A—C13A—H13B	108.5	C17B—C16B—C15B	116.9 (5)
C14A—C13A—H13B	108.5	C17B—C16B—C24B	119.9 (5)
H13A—C13A—H13B	107.5	C15B—C16B—C24B	123.1 (5)
C19A—C14A—C15A	119.2 (4)	C16B—C17B—C18B	124.7 (5)
C19A—C14A—C13A	118.1 (4)	C16B—C17B—H17B	117.7
C15A—C14A—C13A	122.7 (4)	C18B—C17B—H17B	117.7
O1A—C15A—C16A	121.4 (4)	C19B—C18B—C17B	116.1 (5)
O1A—C15A—C14A	118.0 (4)	C19B—C18B—C20B	119.9 (5)
C16A—C15A—C14A	120.6 (4)	C17B—C18B—C20B	123.8 (5)
C15A—C16A—C17A	116.3 (5)	C18B—C19B—C14B	123.1 (5)
C15A—C16A—C24A	123.8 (4)	C18B—C19B—H19B	118.4
C17A—C16A—C24A	119.8 (4)	C14B—C19B—H19B	118.4
C18A—C17A—C16A	124.7 (5)	C21B—C20B—C22B	109.9 (6)
C18A—C17A—H17A	117.6	C21B—C20B—C23B	106.7 (5)
C16A—C17A—H17A	117.6	C22B—C20B—C23B	108.2 (6)
C17A—C18A—C19A	116.7 (5)	C21B—C20B—C18B	112.9 (5)
C17A—C18A—C20A	123.5 (5)	C22B—C20B—C18B	109.0 (5)
C19A—C18A—C20A	119.8 (5)	C23B—C20B—C18B	110.1 (5)
C18A—C19A—C14A	121.6 (5)	C20B—C21B—H21D	109.5
C18A—C19A—H19A	119.2	C20B—C21B—H21E	109.5
C14A—C19A—H19A	119.2	H21D—C21B—H21E	109.5
C22A—C20A—C18A	110.3 (5)	C20B—C21B—H21F	109.5
C22A—C20A—C23A	109.0 (5)	H21D—C21B—H21F	109.5
C18A—C20A—C23A	112.6 (5)	H21E—C21B—H21F	109.5
C22A—C20A—C21A	108.9 (6)	C20B—C22B—H22D	109.5
C18A—C20A—C21A	108.9 (5)	C20B—C22B—H22E	109.5
C23A—C20A—C21A	107.0 (5)	H22D—C22B—H22E	109.5
C20A—C21A—H21A	109.5	C20B—C22B—H22F	109.5
C20A—C21A—H21B	109.5	H22D—C22B—H22F	109.5
H21A—C21A—H21B	109.5	H22E—C22B—H22F	109.5
C20A—C21A—H21C	109.5	C20B—C23B—H23D	109.5
H21A—C21A—H21C	109.5	C20B—C23B—H23E	109.5
H21B—C21A—H21C	109.5	H23D—C23B—H23E	109.5
C20A—C22A—H22A	109.5	C20B—C23B—H23F	109.5
C20A—C22A—H22B	109.5	H23D—C23B—H23F	109.5
H22A—C22A—H22B	109.5	H23E—C23B—H23F	109.5
C20A—C22A—H22C	109.5	C26B—C24B—C27B	110.5 (5)
H22A—C22A—H22C	109.5	C26B—C24B—C25B	107.5 (4)
H22B—C22A—H22C	109.5	C27B—C24B—C25B	106.8 (5)
C20A—C23A—H23A	109.5	C26B—C24B—C16B	111.5 (4)
C20A—C23A—H23B	109.5	C27B—C24B—C16B	109.1 (4)
H23A—C23A—H23B	109.5	C25B—C24B—C16B	111.3 (5)
C20A—C23A—H23C	109.5	C24B—C25B—H25D	109.5
H23A—C23A—H23C	109.5	C24B—C25B—H25E	109.5
H23B—C23A—H23C	109.5	H25D—C25B—H25E	109.5
C26A—C24A—C27A	109.8 (4)	C24B—C25B—H25F	109.5
C26A—C24A—C16A	111.3 (4)	H25D—C25B—H25F	109.5
C27A—C24A—C16A	109.8 (4)	H25E—C25B—H25F	109.5
C26A—C24A—C25A	106.6 (5)	C24B—C26B—H26D	109.5
C27A—C24A—C25A	106.6 (4)	C24B—C26B—H26E	109.5
C16A—C24A—C25A	112.6 (4)	H26D—C26B—H26E	109.5
C24A—C25A—H25A	109.5	C24B—C26B—H26F	109.5
C24A—C25A—H25B	109.5	H26D—C26B—H26F	109.5
H25A—C25A—H25B	109.5	H26E—C26B—H26F	109.5
C24A—C25A—H25C	109.5	C24B—C27B—H27D	109.5
H25A—C25A—H25C	109.5	C24B—C27B—H27E	109.5
H25B—C25A—H25C	109.5	H27D—C27B—H27E	109.5
C24A—C26A—H26A	109.5	C24B—C27B—H27F	109.5
C24A—C26A—H26B	109.5	H27D—C27B—H27F	109.5
H26A—C26A—H26B	109.5	H27E—C27B—H27F	109.5
C24A—C26A—H26C	109.5	Si1B—C28B—H28D	109.5
H26A—C26A—H26C	109.5	Si1B—C28B—H28E	109.5
H26B—C26A—H26C	109.5	H28D—C28B—H28E	109.5
C24A—C27A—H27A	109.5	Si1B—C28B—H28F	109.5
C24A—C27A—H27B	109.5	H28D—C28B—H28F	109.5
H27A—C27A—H27B	109.5	H28E—C28B—H28F	109.5
C24A—C27A—H27C	109.5	Si1B—C29B—H29D	109.5
H27A—C27A—H27C	109.5	Si1B—C29B—H29E	109.5
H27B—C27A—H27C	109.5	H29D—C29B—H29E	109.5
Si1A—C28A—H28A	109.5	Si1B—C29B—H29F	109.5
Si1A—C28A—H28B	109.5	H29D—C29B—H29F	109.5
H28A—C28A—H28B	109.5	H29E—C29B—H29F	109.5
Si1A—C28A—H28C	109.5	Si1B—C30B—H30D	109.5
H28A—C28A—H28C	109.5	Si1B—C30B—H30E	109.5
H28B—C28A—H28C	109.5	H30D—C30B—H30E	109.5
Si1A—C29A—H29A	109.5	Si1B—C30B—H30F	109.5
Si1A—C29A—H29B	109.5	H30D—C30B—H30F	109.5
H29A—C29A—H29B	109.5	H30E—C30B—H30F	109.5
Si1A—C29A—H29C	109.5	S1B—C31B—H31D	109.5
H29A—C29A—H29C	109.5	S1B—C31B—H31E	109.5
H29B—C29A—H29C	109.5	H31D—C31B—H31E	109.5
Si1A—C30A—H30A	109.5	S1B—C31B—H31F	109.5
Si1A—C30A—H30B	109.5	H31D—C31B—H31F	109.5
H30A—C30A—H30B	109.5	H31E—C31B—H31F	109.5
Si1A—C30A—H30C	109.5	S1B—C32B—H32D	109.5
H30A—C30A—H30C	109.5	S1B—C32B—H32E	109.5
H30B—C30A—H30C	109.5	H32D—C32B—H32E	109.5
S1A—C31A—H31A	109.5	S1B—C32B—H32F	109.5
S1A—C31A—H31B	109.5	H32D—C32B—H32F	109.5
H31A—C31A—H31B	109.5	H32E—C32B—H32F	109.5
S1A—C31A—H31C	109.5	Cl2C—C1C—Cl1C	113.6 (3)
H31A—C31A—H31C	109.5	Cl2C—C1C—H1C1	108.9
H31B—C31A—H31C	109.5	Cl1C—C1C—H1C1	108.9
S1A—C32A—H32A	109.5	Cl2C—C1C—H1C2	108.9
S1A—C32A—H32B	109.5	Cl1C—C1C—H1C2	108.9
H32A—C32A—H32B	109.5	H1C1—C1C—H1C2	107.7
S1A—C32A—H32C	109.5	Cl2D—C1D—Cl1D	114.3 (4)
H32A—C32A—H32C	109.5	Cl2D—C1D—H1D1	108.7
H32B—C32A—H32C	109.5	Cl1D—C1D—H1D1	108.7
N1B—Ru1B—N2B	95.09 (15)	Cl2D—C1D—H1D2	108.7
N1B—Ru1B—N3B	78.23 (15)	Cl1D—C1D—H1D2	108.7
N2B—Ru1B—N3B	80.17 (15)	H1D1—C1D—H1D2	107.6
N1B—Ru1B—S1B	90.67 (11)	Cl2E—C1E—Cl1E	114.2 (5)
N2B—Ru1B—S1B	173.35 (11)	Cl2E—C1E—H1E1	108.7
N3B—Ru1B—S1B	97.80 (10)	Cl1E—C1E—H1E1	108.7
N1B—Ru1B—Cl2B	93.91 (12)	Cl2E—C1E—H1E2	108.7
N2B—Ru1B—Cl2B	93.62 (12)	Cl1E—C1E—H1E2	108.7
N3B—Ru1B—Cl2B	169.41 (10)	H1E1—C1E—H1E2	107.6
			
N1A—Ru1A—S1A—O2A	90.25 (19)	N1B—Ru1B—S1B—O2B	−93.0 (2)
N2A—Ru1A—S1A—O2A	−62.1 (11)	N2B—Ru1B—S1B—O2B	57.0 (10)
N3A—Ru1A—S1A—O2A	13.23 (18)	N3B—Ru1B—S1B—O2B	−14.8 (2)
Cl2A—Ru1A—S1A—O2A	−175.14 (16)	Cl2B—Ru1B—S1B—O2B	173.09 (17)
Cl1A—Ru1A—S1A—O2A	−86.11 (16)	Cl1B—Ru1B—S1B—O2B	82.88 (17)
N1A—Ru1A—S1A—C32A	−145.1 (2)	N1B—Ru1B—S1B—C32B	141.9 (2)
N2A—Ru1A—S1A—C32A	62.6 (11)	N2B—Ru1B—S1B—C32B	−68.0 (10)
N3A—Ru1A—S1A—C32A	137.9 (2)	N3B—Ru1B—S1B—C32B	−139.8 (2)
Cl2A—Ru1A—S1A—C32A	−50.49 (19)	Cl2B—Ru1B—S1B—C32B	48.03 (19)
Cl1A—Ru1A—S1A—C32A	38.54 (19)	Cl1B—Ru1B—S1B—C32B	−42.18 (19)
N1A—Ru1A—S1A—C31A	−34.4 (2)	N1B—Ru1B—S1B—C31B	31.8 (2)
N2A—Ru1A—S1A—C31A	173.2 (11)	N2B—Ru1B—S1B—C31B	−178.2 (10)
N3A—Ru1A—S1A—C31A	−111.4 (2)	N3B—Ru1B—S1B—C31B	110.0 (2)
Cl2A—Ru1A—S1A—C31A	60.20 (19)	Cl2B—Ru1B—S1B—C31B	−62.1 (2)
Cl1A—Ru1A—S1A—C31A	149.22 (19)	Cl1B—Ru1B—S1B—C31B	−152.3 (2)
C30A—Si1A—O1A—C15A	−110.8 (4)	C30B—Si1B—O1B—C15B	115.5 (4)
C28A—Si1A—O1A—C15A	9.6 (5)	C28B—Si1B—O1B—C15B	−5.7 (5)
C29A—Si1A—O1A—C15A	134.1 (4)	C29B—Si1B—O1B—C15B	−129.4 (4)
N2A—Ru1A—N1A—C1A	−83.3 (4)	N2B—Ru1B—N1B—C1B	82.5 (4)
N3A—Ru1A—N1A—C1A	−160.7 (4)	N3B—Ru1B—N1B—C1B	161.4 (4)
S1A—Ru1A—N1A—C1A	99.3 (4)	S1B—Ru1B—N1B—C1B	−100.8 (4)
Cl2A—Ru1A—N1A—C1A	11.1 (4)	Cl2B—Ru1B—N1B—C1B	−11.5 (4)
Cl1A—Ru1A—N1A—C1A	170.9 (14)	Cl1B—Ru1B—N1B—C1B	172.9 (13)
N2A—Ru1A—N1A—C5A	104.0 (3)	N2B—Ru1B—N1B—C5B	−102.7 (3)
N3A—Ru1A—N1A—C5A	26.6 (3)	N3B—Ru1B—N1B—C5B	−23.8 (3)
S1A—Ru1A—N1A—C5A	−73.4 (3)	S1B—Ru1B—N1B—C5B	74.0 (3)
Cl2A—Ru1A—N1A—C5A	−161.6 (3)	Cl2B—Ru1B—N1B—C5B	163.3 (3)
Cl1A—Ru1A—N1A—C5A	−1.8 (19)	Cl1B—Ru1B—N1B—C5B	−12.3 (17)
N1A—Ru1A—N2A—C8A	−70.1 (3)	N1B—Ru1B—N2B—C12B	−118.2 (4)
N3A—Ru1A—N2A—C8A	5.8 (3)	N3B—Ru1B—N2B—C12B	164.7 (4)
S1A—Ru1A—N2A—C8A	82.1 (12)	S1B—Ru1B—N2B—C12B	91.9 (11)
Cl2A—Ru1A—N2A—C8A	−165.1 (3)	Cl2B—Ru1B—N2B—C12B	−23.9 (4)
Cl1A—Ru1A—N2A—C8A	106.2 (3)	Cl1B—Ru1B—N2B—C12B	65.9 (4)
N1A—Ru1A—N2A—C12A	125.4 (4)	N1B—Ru1B—N2B—C8B	73.1 (3)
N3A—Ru1A—N2A—C12A	−158.7 (4)	N3B—Ru1B—N2B—C8B	−4.0 (3)
S1A—Ru1A—N2A—C12A	−82.4 (12)	S1B—Ru1B—N2B—C8B	−76.8 (11)
Cl2A—Ru1A—N2A—C12A	30.4 (3)	Cl2B—Ru1B—N2B—C8B	167.4 (3)
Cl1A—Ru1A—N2A—C12A	−58.3 (3)	Cl1B—Ru1B—N2B—C8B	−102.7 (3)
N1A—Ru1A—N3A—C6A	−38.5 (3)	N1B—Ru1B—N3B—C6B	36.4 (3)
N2A—Ru1A—N3A—C6A	−137.6 (3)	N2B—Ru1B—N3B—C6B	133.8 (3)
S1A—Ru1A—N3A—C6A	48.0 (3)	S1B—Ru1B—N3B—C6B	−52.6 (3)
Cl2A—Ru1A—N3A—C6A	−84.7 (6)	Cl2B—Ru1B—N3B—C6B	79.1 (7)
Cl1A—Ru1A—N3A—C6A	139.7 (3)	Cl1B—Ru1B—N3B—C6B	−142.7 (2)
N1A—Ru1A—N3A—C13A	−162.1 (3)	N1B—Ru1B—N3B—C7B	−78.2 (3)
N2A—Ru1A—N3A—C13A	98.7 (3)	N2B—Ru1B—N3B—C7B	19.1 (3)
S1A—Ru1A—N3A—C13A	−75.6 (3)	S1B—Ru1B—N3B—C7B	−167.3 (3)
Cl2A—Ru1A—N3A—C13A	151.7 (4)	Cl2B—Ru1B—N3B—C7B	−35.5 (8)
Cl1A—Ru1A—N3A—C13A	16.0 (3)	Cl1B—Ru1B—N3B—C7B	102.6 (3)
N1A—Ru1A—N3A—C7A	75.0 (3)	N1B—Ru1B—N3B—C13B	158.6 (3)
N2A—Ru1A—N3A—C7A	−24.1 (3)	N2B—Ru1B—N3B—C13B	−104.1 (3)
S1A—Ru1A—N3A—C7A	161.5 (2)	S1B—Ru1B—N3B—C13B	69.5 (3)
Cl2A—Ru1A—N3A—C7A	28.9 (7)	Cl2B—Ru1B—N3B—C13B	−158.8 (5)
Cl1A—Ru1A—N3A—C7A	−106.8 (2)	Cl1B—Ru1B—N3B—C13B	−20.6 (3)
C5A—N1A—C1A—C2A	2.0 (8)	C5B—N1B—C1B—C2B	−2.7 (7)
Ru1A—N1A—C1A—C2A	−170.5 (4)	Ru1B—N1B—C1B—C2B	171.9 (3)
N1A—C1A—C2A—C3A	0.2 (9)	N1B—C1B—C2B—C3B	1.2 (7)
C1A—C2A—C3A—C4A	−2.4 (9)	C1B—C2B—C3B—C4B	1.0 (7)
C2A—C3A—C4A—C5A	2.5 (8)	C2B—C3B—C4B—C5B	−1.5 (7)
C1A—N1A—C5A—C4A	−1.9 (7)	C1B—N1B—C5B—C4B	2.2 (6)
Ru1A—N1A—C5A—C4A	171.5 (4)	Ru1B—N1B—C5B—C4B	−173.1 (3)
C1A—N1A—C5A—C6A	178.4 (4)	C1B—N1B—C5B—C6B	−179.9 (4)
Ru1A—N1A—C5A—C6A	−8.2 (5)	Ru1B—N1B—C5B—C6B	4.8 (5)
C3A—C4A—C5A—N1A	−0.3 (8)	C3B—C4B—C5B—N1B	−0.1 (7)
C3A—C4A—C5A—C6A	179.4 (5)	C3B—C4B—C5B—C6B	−177.8 (4)
C13A—N3A—C6A—C5A	168.6 (4)	C7B—N3B—C6B—C5B	70.7 (4)
C7A—N3A—C6A—C5A	−69.5 (5)	C13B—N3B—C6B—C5B	−166.4 (3)
Ru1A—N3A—C6A—C5A	44.1 (4)	Ru1B—N3B—C6B—C5B	−43.8 (4)
N1A—C5A—C6A—N3A	−25.5 (6)	N1B—C5B—C6B—N3B	27.7 (5)
C4A—C5A—C6A—N3A	154.8 (5)	C4B—C5B—C6B—N3B	−154.4 (4)
C6A—N3A—C7A—C8A	150.5 (4)	C6B—N3B—C7B—C8B	−143.7 (4)
C13A—N3A—C7A—C8A	−86.4 (4)	C13B—N3B—C7B—C8B	92.8 (5)
Ru1A—N3A—C7A—C8A	38.3 (4)	Ru1B—N3B—C7B—C8B	−31.5 (4)
C12A—N2A—C8A—C9A	−1.1 (7)	C12B—N2B—C8B—C9B	0.6 (7)
Ru1A—N2A—C8A—C9A	−166.4 (3)	Ru1B—N2B—C8B—C9B	170.0 (4)
C12A—N2A—C8A—C7A	−179.4 (4)	C12B—N2B—C8B—C7B	177.4 (4)
Ru1A—N2A—C8A—C7A	15.3 (5)	Ru1B—N2B—C8B—C7B	−13.1 (5)
N3A—C7A—C8A—N2A	−36.5 (5)	N3B—C7B—C8B—N2B	30.5 (6)
N3A—C7A—C8A—C9A	145.2 (4)	N3B—C7B—C8B—C9B	−152.7 (4)
N2A—C8A—C9A—C10A	0.6 (7)	N2B—C8B—C9B—C10B	0.3 (7)
C7A—C8A—C9A—C10A	178.8 (4)	C7B—C8B—C9B—C10B	−176.3 (5)
C8A—C9A—C10A—C11A	0.3 (7)	C8B—C9B—C10B—C11B	−1.2 (8)
C9A—C10A—C11A—C12A	−0.6 (7)	C9B—C10B—C11B—C12B	1.2 (8)
C8A—N2A—C12A—C11A	0.8 (7)	C8B—N2B—C12B—C11B	−0.6 (7)
Ru1A—N2A—C12A—C11A	164.8 (3)	Ru1B—N2B—C12B—C11B	−169.0 (4)
C10A—C11A—C12A—N2A	0.0 (7)	C10B—C11B—C12B—N2B	−0.3 (8)
C6A—N3A—C13A—C14A	59.5 (5)	C6B—N3B—C13B—C14B	−53.5 (5)
C7A—N3A—C13A—C14A	−60.2 (5)	C7B—N3B—C13B—C14B	67.6 (5)
Ru1A—N3A—C13A—C14A	179.0 (3)	Ru1B—N3B—C13B—C14B	−171.1 (3)
N3A—C13A—C14A—C19A	−90.4 (5)	N3B—C13B—C14B—C19B	84.7 (5)
N3A—C13A—C14A—C15A	87.5 (5)	N3B—C13B—C14B—C15B	−94.8 (6)
Si1A—O1A—C15A—C16A	−97.0 (5)	Si1B—O1B—C15B—C14B	−79.7 (6)
Si1A—O1A—C15A—C14A	84.5 (5)	Si1B—O1B—C15B—C16B	102.6 (5)
C19A—C14A—C15A—O1A	−172.9 (4)	C19B—C14B—C15B—O1B	174.8 (4)
C13A—C14A—C15A—O1A	9.2 (7)	C13B—C14B—C15B—O1B	−5.7 (7)
C19A—C14A—C15A—C16A	8.5 (7)	C19B—C14B—C15B—C16B	−7.4 (7)
C13A—C14A—C15A—C16A	−169.4 (4)	C13B—C14B—C15B—C16B	172.0 (4)
O1A—C15A—C16A—C17A	173.0 (4)	O1B—C15B—C16B—C17B	−176.8 (4)
C14A—C15A—C16A—C17A	−8.5 (7)	C14B—C15B—C16B—C17B	5.6 (7)
O1A—C15A—C16A—C24A	−9.7 (7)	O1B—C15B—C16B—C24B	1.7 (7)
C14A—C15A—C16A—C24A	168.8 (4)	C14B—C15B—C16B—C24B	−176.0 (4)
C15A—C16A—C17A—C18A	1.3 (7)	C15B—C16B—C17B—C18B	−0.9 (8)
C24A—C16A—C17A—C18A	−176.1 (5)	C24B—C16B—C17B—C18B	−179.4 (5)
C16A—C17A—C18A—C19A	5.7 (8)	C16B—C17B—C18B—C19B	−1.8 (8)
C16A—C17A—C18A—C20A	−177.3 (5)	C16B—C17B—C18B—C20B	−177.4 (5)
C17A—C18A—C19A—C14A	−5.7 (7)	C17B—C18B—C19B—C14B	−0.2 (8)
C20A—C18A—C19A—C14A	177.1 (5)	C20B—C18B—C19B—C14B	175.6 (5)
C15A—C14A—C19A—C18A	−1.1 (7)	C15B—C14B—C19B—C18B	4.8 (8)
C13A—C14A—C19A—C18A	176.9 (4)	C13B—C14B—C19B—C18B	−174.7 (4)
C17A—C18A—C20A—C22A	−115.9 (6)	C19B—C18B—C20B—C21B	177.0 (5)
C19A—C18A—C20A—C22A	61.1 (7)	C17B—C18B—C20B—C21B	−7.5 (8)
C17A—C18A—C20A—C23A	6.1 (8)	C19B—C18B—C20B—C22B	−60.6 (7)
C19A—C18A—C20A—C23A	−176.9 (5)	C17B—C18B—C20B—C22B	114.9 (6)
C17A—C18A—C20A—C21A	124.6 (6)	C19B—C18B—C20B—C23B	57.9 (7)
C19A—C18A—C20A—C21A	−58.4 (7)	C17B—C18B—C20B—C23B	−126.6 (6)
C15A—C16A—C24A—C26A	−42.8 (6)	C17B—C16B—C24B—C26B	−136.2 (5)
C17A—C16A—C24A—C26A	134.5 (5)	C15B—C16B—C24B—C26B	45.4 (7)
C15A—C16A—C24A—C27A	79.0 (6)	C17B—C16B—C24B—C27B	101.4 (6)
C17A—C16A—C24A—C27A	−103.8 (5)	C15B—C16B—C24B—C27B	−77.0 (6)
C15A—C16A—C24A—C25A	−162.4 (5)	C17B—C16B—C24B—C25B	−16.1 (7)
C17A—C16A—C24A—C25A	14.8 (7)	C15B—C16B—C24B—C25B	165.4 (5)
